# Taurine represses age‐associated gut hyperplasia in *Drosophila* via counteracting endoplasmic reticulum stress

**DOI:** 10.1111/acel.13319

**Published:** 2021-02-09

**Authors:** Gang Du, Zhiming Liu, Zihua Yu, Zhangpeng Zhuo, Yuedan Zhu, Juanyu Zhou, Yang Li, Haiyang Chen

**Affiliations:** ^1^ Laboratory for Stem Cell and anti‐Aging Research National Clinical Research Center for Geriatrics West China Hospital Sichuan University Chengdu China; ^2^ Key Laboratory of Gene Engineering of the Ministry of Education State Key Laboratory of Biocontrol School of Life Sciences Sun Yat‐sen University Guangzhou China

**Keywords:** aging, ER stress, gut, intestinal stem cell, JNK signaling, taurine, unfolded protein response

## Abstract

As they age, adult stem cells become more prone to functional decline, which is responsible for aging‐associated tissue degeneration and diseases. One goal of aging research is to identify drugs that can repair age‐associated tissue degeneration. Multiple organ development‐related signaling pathways have recently been demonstrated to have functions in tissue homeostasis and aging process. Therefore, in this study, we tested several chemicals that are essential for organ development to assess their ability to delay intestinal stem cell (ISC) aging and promote gut function in adult *Drosophila*. We found that taurine, a free amino acid that supports neurological development and tissue metabolism in humans, represses ISC hyperproliferation and restrains the intestinal functional decline seen in aged animals. We found that taurine represses age‐associated ISC hyperproliferation through a mechanism that eliminated endoplasmic reticulum (ER) stress by upregulation of the target genes of unfolded protein response in the ER (UPR^ER^) and inhibiting the c‐Jun N‐terminal kinase (JNK) signaling. Our findings show that taurine plays a critical role in delaying the aging process in stem cells and suggest that it may be used as a natural compound for the treatment of age‐associated, or damage‐induced intestinal dysfunction in humans.

## Graphical abstract

In young *Drosophila*, taurine activates the UPRER target genes in ISCs, which in turn attenuates ER stress and prevents the over‐activation of JNK signaling. When flies get old, the abundance of taurine dramatically reduces, which causes an increase in ER stress, and the activation of UPRER sensors in ISCs. Ultimately, this leads to the hyperproliferation of ISCs with age.

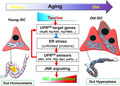

## 1 INTRODUCTION

The aging process is characterized by a progressive decline in the normal functions of tissues and organs (Lopez‐Otin et al., 2013), making individuals much more sensitive to diverse stimuli, such as pathogens, toxins, and chemical or mechanical injury (Deleidi et al., 2015), and leading to an increased vulnerability to disease and death. In recent years, numerous biologic phenomena have been linked to the aging process, including stem cell exhaustion, which is regarded as one of the most important hallmarks of organismal aging (Lopez‐Otin et al., 2013). Many studies have demonstrated that adult stem cells exhibit dysfunction or exhaustion during aging (Ambrosi et al., 2017). When stem cells are forced to activate repeatedly, they can become exhausted and eventually fail to accomplish tissue turnover (Haller et al., 2017). When adult stem cells can avoid aging‐related exhaustion, the functional decline of organs can be significantly delayed (Gervais & Bardin, 2017). Preventing stem cell exhaustion is therefore a promising area of anti‐aging research; however, the underlying mechanisms of aging‐related functional decline in adult stem cells remain unclear due to the complexity of the stem cell niche, and the highly dynamic nature of the external environment. Identifying small molecular compounds that can improve stem cell function will not only yield drugs that can be used to promote healthy aging but can also shed light on the mechanisms involved in stem cell exhaustion.

To date, the underlying mechanisms affecting organismal development have been, to a large extent, uncovered. Intriguingly, recent studies have shown that many of the signaling pathways that regulate organ development also play important roles in the maintenance of tissue homeostasis and/or the aging‐associated functional decline of organs (Campisi et al., 2019). This indicates that aging shares, at least in part, similar mechanisms with organismal development. Therefore, it will be of great interest to determine whether essential nutrients that have been reported to modulate these signaling pathways and promote organ development can also exhibit anti‐aging effects.

In mammals, taurine is a conditionally essential beta‐amino acid derived from cysteine which needs to be supplemented by dietary sources (Ripps & Shen, 2012). It has been reported that taurine is crucial for multiple organ development (Rera et al., 2012). Recent studies have revealed that taurine can be used to treat aging‐associated diseases in the heart, skin, skeletal muscle, and liver (Miyazaki & Matsuzaki, 2014). Based on these studies, we postulate that taurine may also regulate adult stem cell functions. Taurine administration has already been reported to significantly stimulate neural stem cell proliferation and differentiation (Wang et al., 2015); however, it remains unclear whether taurine can play a role in preventing stem cell exhaustion and thus has an anti‐aging effect.

**FIGURE 1:**
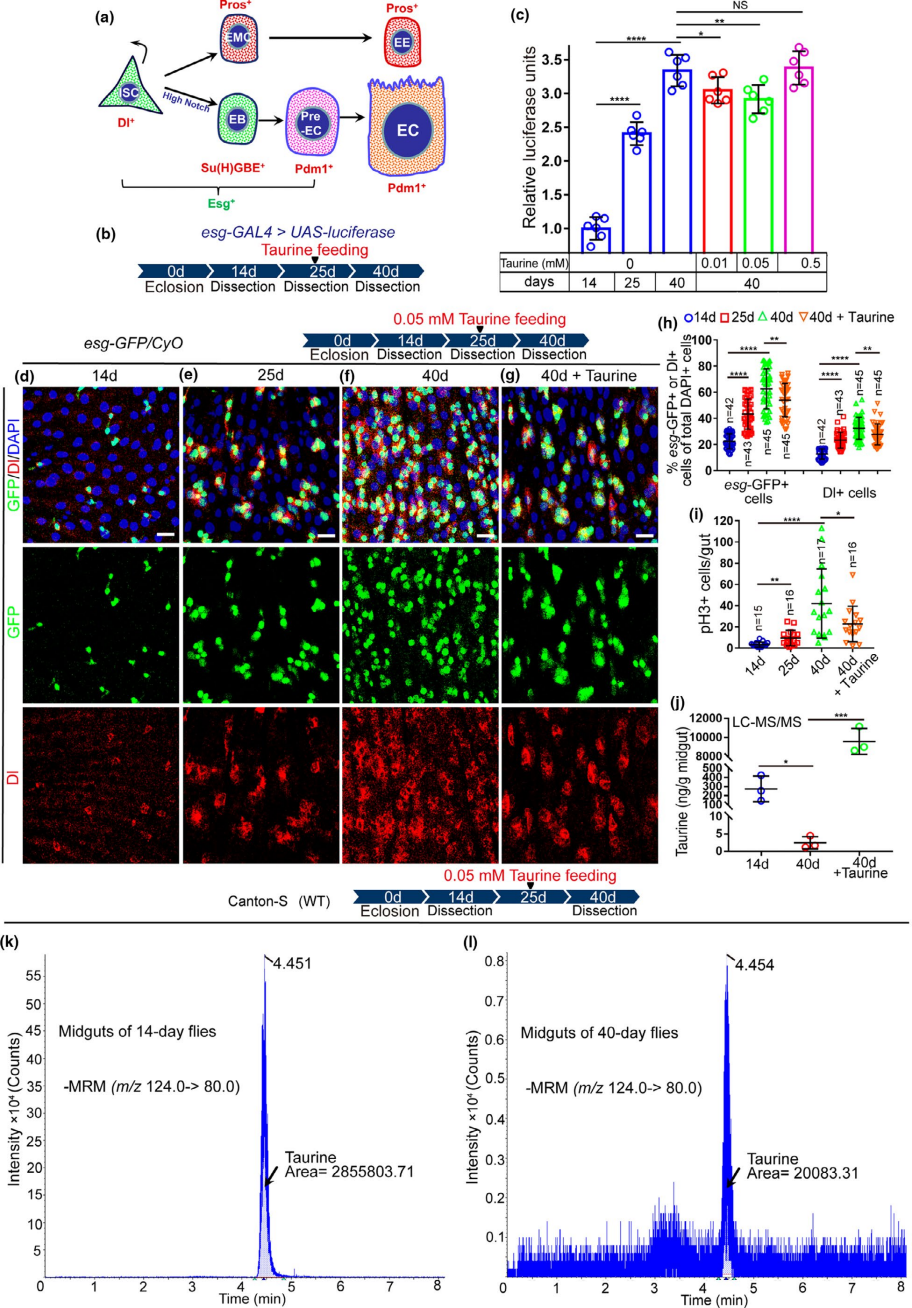
Taurine supplementation represses ISC hyperproliferation in aged *Drosophila*. (a) Schematic diagram of cell types and markers in the *Drosophila* midgut. (b) A representative schematic of the *Drosophila* “esg > luciferase” reporter system. (c) Quantification of luciferase activity of midguts of 14‐, 25‐, and 40‐day flies with or without taurine supplementation. Error bars show the standard deviation (SD) of six independent experiments. (d–g) Midguts were dissected at 14, 25, 40, and 40 days with taurine treatment and stained with Dl (red; ISC marker), and *esg*‐GFP (green; EB and ISC marker). (h, i) The average numbers of *esg*‐GFP^+^, Dl^+^, and pH3^+^ cells are significantly lower in 40‐day midguts after taurine supplementation. (j) Quantification of the content of taurine in midguts of 14‐day, 40‐day, and 40‐day flies fed with taurine using LC‐ESI‐MS/MS. Error bars indicate the SD of three independent experiments. (k, i) LC‐MS chromatograms of taurine in midguts of 14‐day (k) and 40‐day (i) flies. Data information: Scale bars represent 10 µm. DAPI‐stained nuclei are shown in blue. Error bars represent SDs. Student's *t* tests, *p < 0.05, **p < 0.01, ***p < 0.001, ****p < 0.0001. non‐significance (NS) represents *p* > 0.05. See also Figure S1

The *Drosophila* midgut is a widely used model system to study stem cell function because of its simple cellular components and the wide array of tools that exist for genetic manipulation in *Drosophila* midgut. It can be used to identify potential strategies to enhance the regenerative capacity of adult stem cells and to uncover the underlying mechanisms of stem cell exhaustion and any anti‐aging effects. *Drosophila* intestinal stem cells (ISCs) expressing Notch ligand Delta (Dl) and transcription factor Escargot (Esg) reside in the basement membrane of the digestive tract (Miguel‐Aliaga et al., 2018). ISCs are responsible for the rapid turnover of the midgut epithelium and divide to self‐renew or differentiate into progenitor cells (either enteroblasts (EBs) or enteroendocrine mother cells (EMCs), depending on Notch activity), which will further differentiate into absorptive enterocytes (ECs) or enteroendocrine cells (EEs) (Figure 1a). The number of stem and progenitor cells is relatively small and is stable in young and homeostatic midguts but increases sharply during aging (Biteau et al., 2008). Several signaling pathways, such as c‐Jun N‐terminal kinase (JNK) (Biteau et al., 2008), mTOR (Johnson et al., 2013), and ROS (Chen et al., 2017), are involved in these aging‐associated alterations. These signaling pathways affect the precise division and differentiation of ISCs and their dysregulation could disrupt the intestinal barrier and the acid–base balance of the digestive tract (Li et al., 2016). Recent studies have revealed that preventing ISC hyperproliferation (either by genetic manipulation or drug administration) can significantly increase *Drosophila* lifespan (Gervais & Bardin, 2017; Wang et al., 2015). Therefore, *Drosophila* midgut is an ideal model for us to use in this study to examine whether taurine affects stem cell behavior and aging.

In this study, we show that taurine supplementation can attenuate age‐associated hyperplasia in the *Drosophila* intestine and promote activation of the unfolded protein response in the endoplasmic reticulum (UPR^ER^) in intestinal stem cells and subsequently suppresses JNK signaling and ISC hyperproliferation. Our findings provide new insights into the underlying mechanisms influencing stem cell aging and a potential anti‐aging therapy.

## 2 RESULTS

### 2.1 Taurine supplementation represses the elevated ISC hyperproliferation seen in aged *Drosophila*

In *Drosophila* midguts, ISC proliferation rate markedly increases upon aging (Choi et al., 2008; Cui et al., 2019) as shown by the continuous accumulation of *escargot* (*esg*)‐positive (*esg* ^+^) cells (ISCs and EBs) and Phospho‐Histone 3‐positive (pH3^+^) cells (pH3 only stains mitotic cells (ISCs) in *Drosophila* midguts). Recent studies have shown that many organ development‐related signaling pathways can regulate tissue homeostasis and aging (Choi et al., 2008; Cui et al., 2019). We, therefore, want to test whether some small molecule compounds that are essential for organ development can prevent aging‐related ISC hyperproliferation in *Drosophila* midguts using an “*esg*‐luciferase” reporter system (Figure 1b) which allows real‐time tracking and quantification of changes in *esg* ^+^ cells.

We found that taurine (a conditionally essential amino acid that can be synthesized in the body and obtained from the diet) significantly repressed the accumulation of *esg* ^+^ cells in aged flies (Figure 1c, Figure S1c). Of the four concentrations tested (0.01 mM, 0.05 mM, 0.5 mM, and 5 mM), 0.05 mM of taurine was most effective at repressing *esg* ^+^ cell accumulation in the midguts of aged flies (Figure 1c, Figure S1c). To further confirm taurine's capacity to prevent ISC hyperproliferation, we used an *esg*‐GFP reporter to visualize ISCs and their differentiating progenies in aged flies. Consistent with previous results, 0.05 mM of taurine significantly delayed the increase of *esg*‐GFP^+^ cells in midguts of flies during aging (Figure 1d–h, Figure S1a). Dl antibody staining was used to label ISCs in aged flies, and the number of Dl^+^ ISCs was significantly lower in flies fed with 0.05 mM of taurine compared with the controls (Figure 1d–h, Figure S1a). The mitotic rate indicated by pH3 staining also showed that taurine supplementation significantly repressed aging‐associated ISC hyperproliferation in *Drosophila* (Figure 1i, Figure S1b). It is important to notice that we found excess taurine consumption did not bring more benefit for anti‐ISC‐Aging but could be harmful to the fly health (Figure 1c, Figure S1d‐f).

### 2.2 Taurine abundance decreases in the midguts of aged flies

Although it can be synthesized from cysteine, diet is the major source of taurine for animals (Luo et al., 2019). To ensure proper organ development, individuals need extra taurine from the daily diet (Jung & Choi, 2019). Previous studies have reported that several diseases, including diabetes, heart failure, muscle and inflammatory diseases, and metabolic diseases, are associated with taurine deficiency (Schaffer & Kim, 2018; Stacchiotti et al., 2018). However, whether taurine deficiency also occurs during intestinal aging remains unknown. Liquid chromatography–electrospray ionization–mass spectrometry (LC‐ESI‐MS/MS) analyses were performed to measure taurine abundance in the midgut of flies of a range of ages. These data indicated that taurine levels in the midguts of older flies were lower than those of younger flies (Figure 1j–l and Figure S1 g–h). Supplementation of taurine can effectively increase the levels of taurine in the midguts of aged flies (Figure 1j and Figure S1i). This suggests that the decrease of taurine may contribute to the functional decline of ISCs.

### 2.3 Taurine administration prevents stimulus‐induced midgut hyperplasia

**FIGURE 2:**
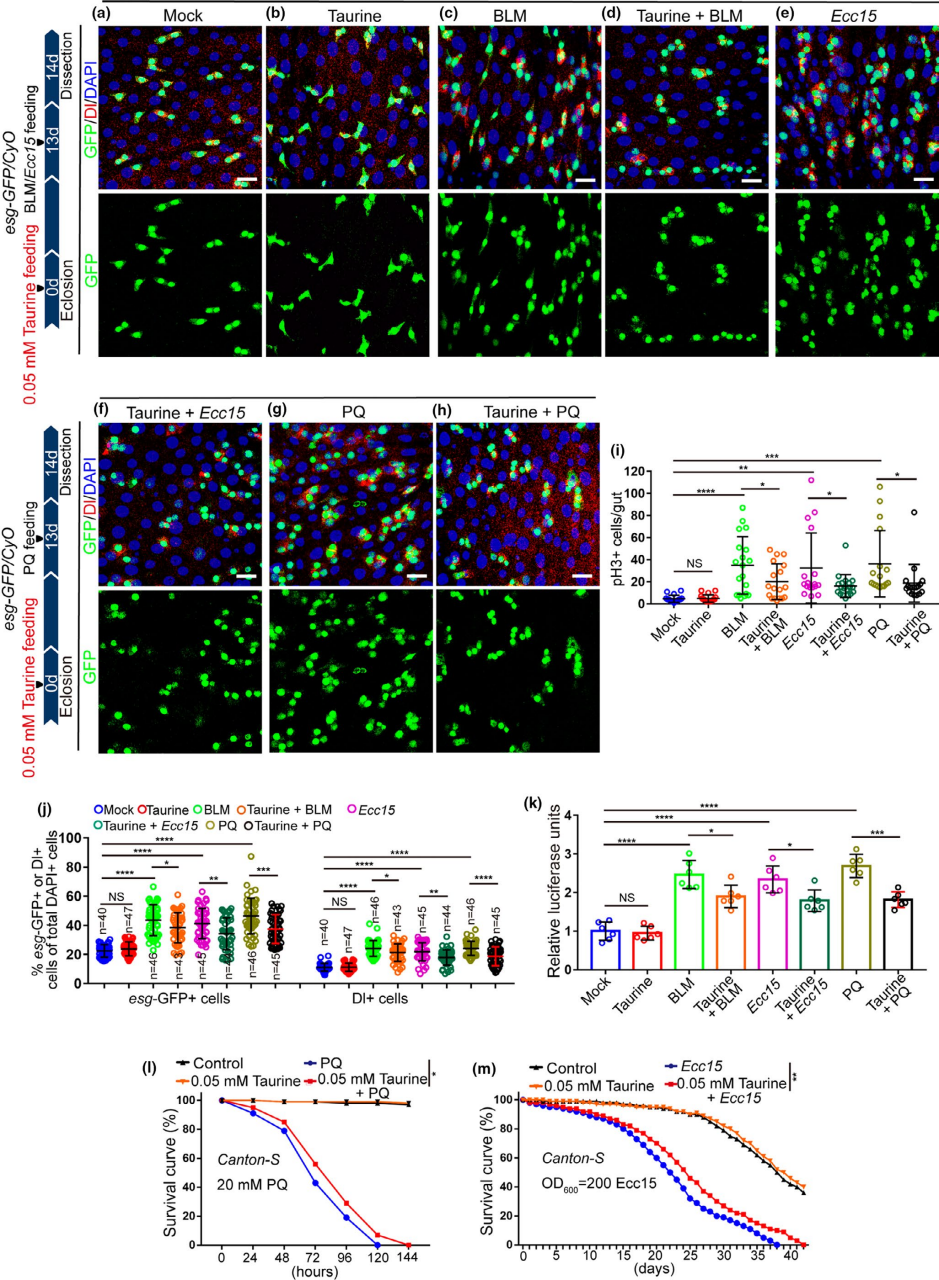
Taurine supplementation prevents stimulus‐induced midgut hyperplasia. (a‐h) Immunofluorescence images of midguts with *esg*‐GFP and Dl staining for Mock (a), Taurine(b), BLM (c), Taurine with BLM (d), *ECC15* (e), Taurine with *ECC15* (f), PQ (g), and Taurine with PQ (h). Mock represents 14‐day midguts without Taurine, BLM, *Ecc15*, or PQ treatment. *esg*‐GFP (green) was used to visualize ISCs and non‐dividing enteroblasts (EBs). Dl staining (red) identifies ISCs. (i, j) The average number of *esg*‐GFP^+^ and Dl^+^ (j), pH3^+^ (i) cells after injury by BLM, *ECC15*, and PQ, with or without taurine supplementation. (k) Quantification of the luciferase activity of midguts of Mock, Taurine, BLM, BLM with taurine, *ECC15*, *ECC15* with taurine, PQ, and PQ with taurine. Data show six independent experiments. (l, m) Survival percentage of flies with and without taurine supplementation under PQ (l) or *ECC15* (m) treatments. 3 independent experiments were conducted. Data information: DAPI stained nuclei are shown in blue. Error bars represent SDs. Scale bars represent 10 µm. For analysis of the statistical significance of differences between two groups, two‐tailed unpaired Student's *t* tests were used. For the survival test, the log‐rank test was used to analyze the statistical significance. *p < 0.05, **p < 0.01, ***p < 0.001, ****p < 0.0001. non‐significance (NS) represents *p* > 0.05

Accumulation of environmental stress‐induced damages is widely considered to be one of the main causes of organismal aging (Lavretsky & Newhouse, 2012; Luo et al., 2020). As aged midguts suffer hyperplasia caused by ISC hyperproliferation, so too do the midguts of young flies when exposed to extracellular stimuli such as oxidizing agents, noxious pathogens, and poisoned foods (Buchon et al., 2009; Hochmuth et al., 2011). We found that taurine supplementation significantly prevented ISC over‐proliferation induced by bleomycin (BLM; causes DNA breaks and genomic instability) and *Erwinia carotovora* (*ECC15*; triggers a systemic immune response) treatments (Figure 2a–f,i–k), and also had some inhibitory effect on midgut hyperplasia induced by paraquat (PQ; induces oxidative stress) (Figure 2a,b,g–k). We then tested the survival rate of flies under chronic damage (induced by continuously feeding with *ECC15* and PQ) and found that supplementation with 0.05 mM taurine significantly increased the survival rate of flies under continual stimuli (Figure 2l–m, and Figure S2a‐b). This demonstrates that taurine increases fly tolerance to noxious environmental stimuli.

### 2.4 Taurine supplementation prevents the aging‐associated decline of intestinal functions in *Drosophila*

**FIGURE 3:**
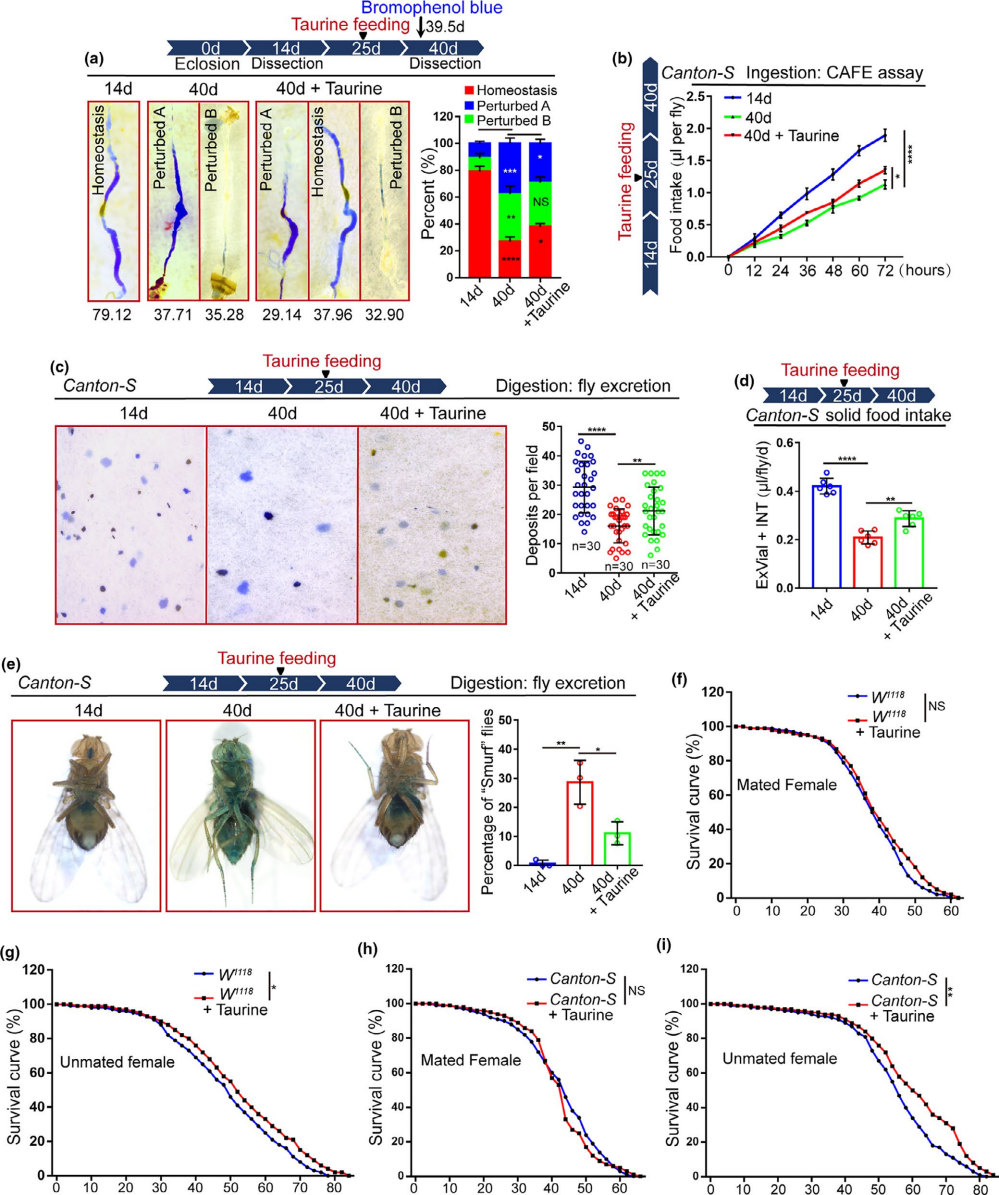
Taurine prevents the age‐related functional decline of the *Drosophila* intestine. (a) Representative imagines and quantification of the percentage of intestinal acid–base homeostasis in flies at 14th and 40th day, with and without taurine. *N* = 90 flies per group. (b) Food intake of flies measured using the CAFÉ assay. Mean ± SD is shown. Error bars show the SD of three independent experiments. (c) Excretion from flies was treated with Bromophenol blue. Representative imagines of deposits and quantification of deposits with indicated treatment are shown. Each sample contains three independent experiments. Excretions are quantified for 30 fields in each group of 12 flies. (d) Measurements of solid food intake in *Drosophila* are shown. Flies of 14‐day, 40‐day, and 40‐day with Taurine supplementation were subjected to solid food in the presence of blue dye. Blue dye was extracted and quantified via spectrophotometry. Data presented as mean + SD, from 6 separate experiments. (e) Representative images and quantification of the percentage of “Smurf” flies from 14‐day, 40‐day, and 40‐day with Taurine supplementation after consuming a non‐absorbed food dye. Each sample contains three independent experiments. (f–g) Survival curves (%) of female (f) and male (i) *W^1118^* flies with and without 0.05 mM of taurine supplementation. 3 independent experiments were conducted. (h–i) Survival curves (%) of female (h) and male (i) *Canton*‐*S* flies with and without 0.05 mM of taurine supplementation. 3 independent experiments were conducted. Data information: Scale bars represent 10 µm. DAPI‐stained nuclei are shown in blue. Error bars represent SDs. Student's t tests, *p < 0.05, **p < 0.01, ***p < 0.001, ****p < 0.0001. non‐significance (NS) represents *p* > 0.05

Previous studies have shown that aging‐associated ISC hyperproliferation in *Drosophila* leads to a remarkable decline in midgut digestive functions, including a decline in both food intake and excretion, and the loss of gastrointestinal acid–base homeostasis (Deshpande et al., 2014; Li et al., 2016). Since taurine supplementation can prevent ISC hyperproliferation in aged flies, we wanted to investigate whether it also retards the decline of intestinal functions in aged flies. In aged flies, 0.05 mM taurine supplementation restrained the deterioration of intestinal acid–base homeostasis (Figure 3a), partially restored the reduction of both food intake and excretion (Figure 3b–d, and Figure S3a‐b), and strengthened the intestinal barrier function (Figure 3e). Moreover, we investigated whether taurine supplementation can prolong lifespan. We found that 0.05 mM of taurine extended the lifespan of flies (Figure 3f–i). We noticed that excess taurine consumption did not extend but shorten the lifespan of flies (Figure S3d), which suggested that the high concentration of taurine might be harmful to the health of flies. These results show that 0.05 mM of taurine supplementation effectively prevents the intestinal functional decline induced by aging‐associated ISC hyperproliferation and prolong *Drosophila* lifespan.

### 2.5 Taurine supplementation induces the activation of the UPR^ER^ target genes in the midguts of aged *Drosophila*

**FIGURE 4:**
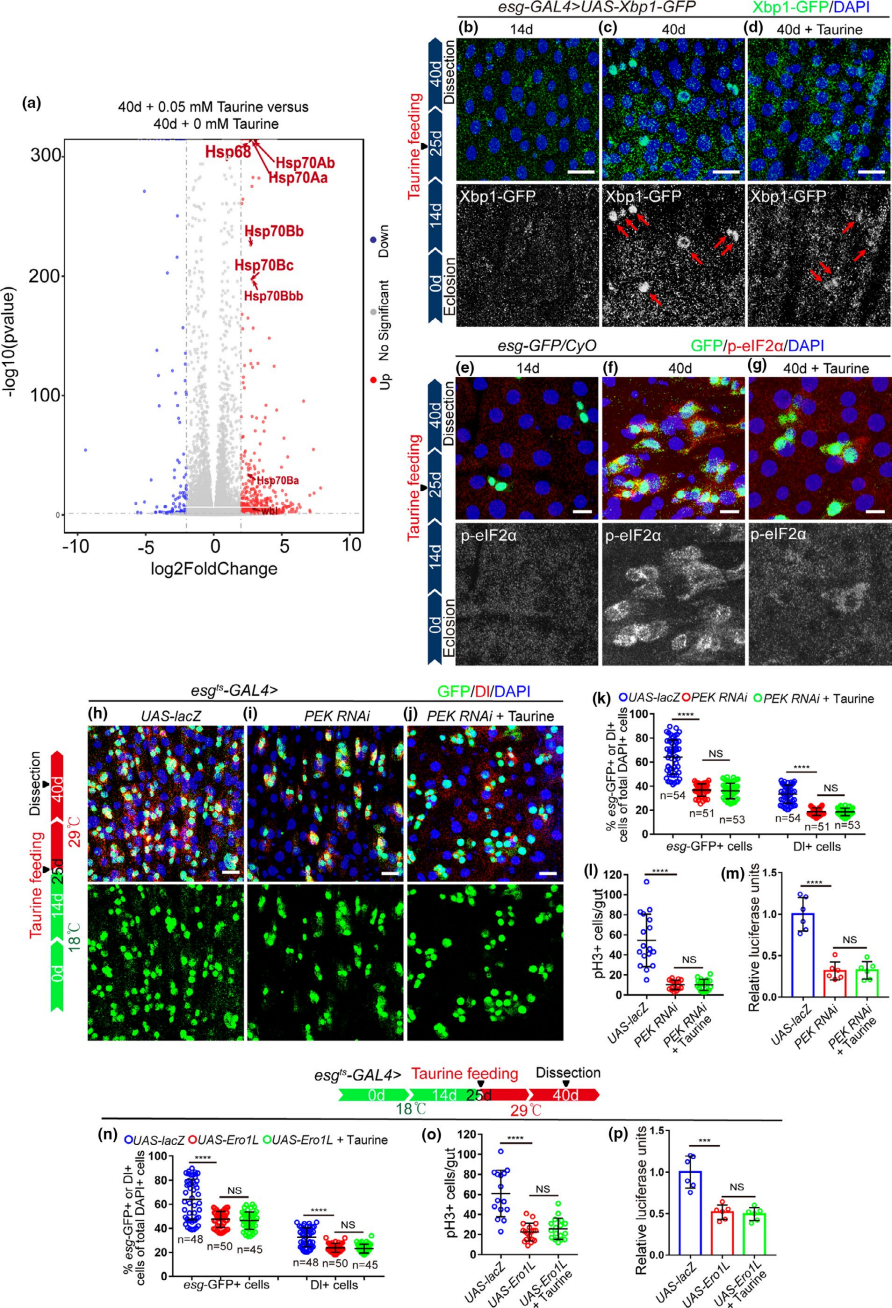
Taurine prevents ISC aging by promoting the upregulation of UPR^ER^ target genes. (a) Volcano plots of differentially expressed genes compared between 40‐day *Drosophila* with and without 0.05 mM taurine supplementation. Red symbols indicate significantly upregulated genes (Log2 FC > 0.6 and *p* < 0.05). Blue symbols indicate significantly downregulated genes. Gray symbols indicate genes that did not change significantly. (b–d) Representative imagines of Xbp1‐GFP staining of midguts of 14‐day flies, 40‐day flies, and 40‐day flies with taurine treatment. Red arrows indicate Xbp1‐GFP‐positive cells. (e–g) Immunofluorescence imagines of *esg*‐GFP and p‐eIF2α staining from 14‐day flies, 40‐day flies, and 40‐day flies with taurine treatment. (h–j) Representative imagines of *esg*‐GFP stating of control flies, *PEK* RNAi flies, and *PEK* RNAi flies with 0.05 mM taurine supplementation. (k–l) Quantification of the number of *esg*‐GFP‐positive, Dl‐positive, and pH3+ cells of control flies, *PEK* RNAi flies, and *PEK* RNAi flies with 0.05 mM taurine supplementation. (m) Quantification of luciferase activity of midguts with indicated genotypes and treatment. Error bars show the standard deviation (SD) of six independent experiments. (n–o) Quantification of the number of *esg*‐GFP‐positive, Dl‐positive, and pH3+ cells of flies with indicated genotypes and treatment. (p) Quantification of luciferase activity of midguts of control flies, *Ero1L*‐overexpressed flies, and *Ero1L*‐overexpressed flies supplemented with 0.05 mM taurine. Error bars show the standard deviation (SD) of six independent experiments. Data information: All data included 3 biological replicates. Error bars represent SDs. Student's *t* tests, *p < 0.05, **p < 0.01, ***p < 0.001, ****p < 0.0001. Non‐significance (NS) represents *p* > 0.05. scale bars represent 10 μm

To identify the mechanisms by which taurine prevent ISC hyperproliferation in old flies, we performed RNA sequencing (RNA‐seq) on dissected midguts of flies both with and without taurine supplementation (Table S1). The results of the RNA‐seq analyses showed that a cluster of the UPR^ER^ target genes (including *Wbl*, *Hsp70Bbb*, *Hsp70Ab*, *Hsp70Bb*, *Hsp68*, *Hsp70Bc*, *Hsp70Ba*, and *Hsp70Aa*) had significantly higher expression levels in the aged midguts (40 days) of *Drosophila* supplemented with taurine compared with those without (Figure 4a, Figure S4c, and Table S2). Kyoto encyclopedia of genes and genomes (KEGG) pathway enrichment analysis of differentially expressed genes, which were only upregulated in old midguts of *Drosophila* given taurine, showed significant enrichment of genes involved in protein processing in the endoplasmic reticulum (ER) (genes in this term mainly play roles in the regulation of ER stress) (Figure S4a,c, and Table S2). The UPR^ER^ is a cellular stress response that is triggered by the ER stress (which occurs when the capacity of the ER to fold proteins becomes overburdened, resulting in the accumulation of unfolded or misfolded proteins) (Hetz, 2012). To counter ER stress, the UPR^ER^ target genes are activated by the UPR^ER^ to reduce the unfolded protein load partially through inducing expression of several stress‐responsive chaperones, such as *HSP70*, *HSP27*, and *HSP90* (Salminen & Kaarniranta, 2010). The UPR^ER^ has been reported to play a key role in regulating aging‐associated ISC hyperproliferation (Hetz, 2012; Wang, Zhu, et al., 2015), and based on these previous studies and current results, we predict that taurine prevents aging‐associated gut hyperplasia via a mechanism that mediated by the UPR^ER^ target genes.

To confirm the results of our RNA‐seq analyses, *esg*‐GFP^+^ cells were sorted to eliminate the interference from other intestinal cell types (Figure S4d) and quantitative reverse transcription‐PCR (qRT‐PCR) analyses were performed on these sorted cells. The qRT‐PCR analyses of selected UPR^ER^ target genes (*Wbl*, *Hsp70*, and *Hsp68*) showed similar expression patterns to the RNA‐seq analysis (Figure S4b). Furthermore, immunostaining of *Xbp1*‐GFP (a reporter for ER stress) and Phosphorylated eIF2α (p‐eIF2α, a widely used biomarker for indicating the status of the ER stress in cells (Rath et al., 2012)) was also significantly downregulated in the ISCs of flies fed with taurine (Figure 4b–g). These findings suggest that taurine probably prevents ISC aging through counteracting ER stress (which was eliminated by the upregulation of the UPR^ER^ target genes) in ISCs of aged flies with taurine treatment.

### 2.6 Taurine prevents ISC aging by eliminating ER stress

**FIGURE 5:**
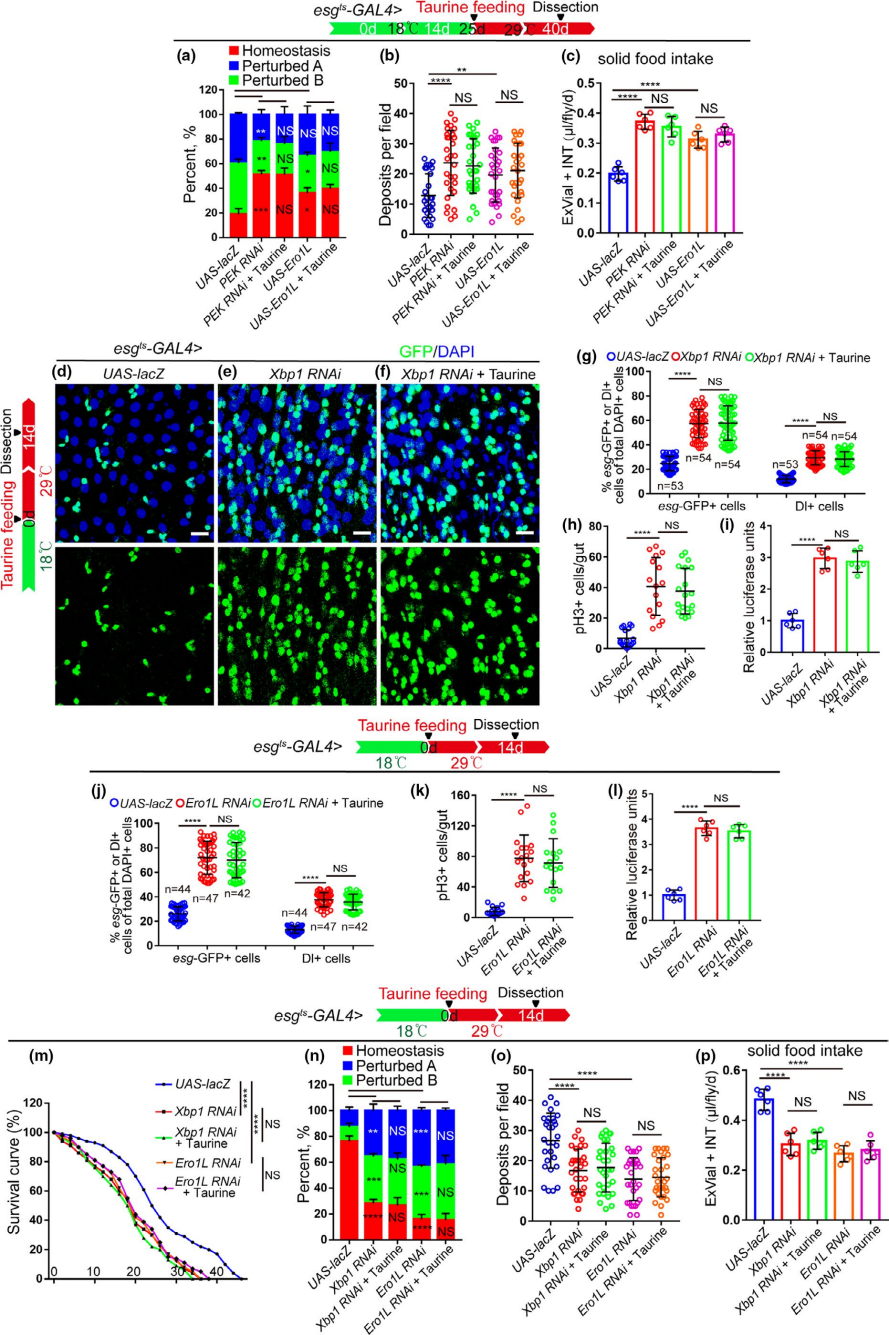
Taurine does not restore the gut hyperplasia phenotype seen in *Drosophila* with an impaired UPR^ER^. (a) Quantification of the percentage of each category of flies with indicated genotypes and treatment. The numbers of midguts from left to right are 90, 90, and 90. Each group contains three independent experiments. (b) Quantification of excretion numbers of flies with indicated genotypes and treatment. (c) Measurements of solid food intake of *Drosophila* are shown. (d–f) Representative immunofluorescence images of midguts with *esg*‐GFP and Dl staining of control flies and *Xbp1*‐depleted flies with or without taurine treatment. (g–h) Quantification of the mean number of *esg*‐GFP^+^, Dl^+^, and pH3^+^ cells of control flies, *Xbp1* RNAi flies, and *Xbp1* RNAi flies fed with 0.05 mM taurine. (i) Quantification of the luciferase activity of midguts of control flies, *Xbp1* RNAi flies, and *Xbp1* RNAi flies with 0.05 mM taurine supplementation. Error bars show the standard deviation (SD) of six independent experiments. (j–l) Quantification of the mean number of *esg*‐GFP^+^, Dl^+^, and pH3^+^ cells (j, k) and the luciferase activity (l) of midguts of control flies, *Ero1L* RNAi flies, and *Ero1L* RNAi flies with 0.05 mM taurine supplementation. (m) Survival curves (%) of indicated genotype with and without Taurine treatment. 3 independent experiments were conducted. (n) Quantification of three categories of midguts treated with the pH indicator Bromophenol Blue in *Drosophila* with indicated genotypes and treatment. The number of quantified midguts from left to right are 90, 90, 90, 90, and 90. Each group contains three independent experiments. (o) Quantifications of deposits with indicated treatment are shown. Each sample contains three independent experiments. Excretions are quantified for 30 fields in each group of 12 flies. (p) Measurements of solid food intake in *Drosophila* are shown. Data information: Scale bars represent 10 µm. DAPI‐stained nuclei are shown in blue. Error bars represent SDs. Student's *t* tests, *p < 0.05, **p < 0.01, ***p < 0.001, ****p < 0.0001. non‐significance (NS) represents *p* > 0.05

Recent studies have shown that the UPR^ER^ plays a central role in regulating intestinal homeostasis in aging organisms (Hetz & Saxena, 2017; Wang et al., 2014; Wang, Zhu, et al., 2015). We found that the increase of *esg*‐GFP^+^ cells, Dl^+^ cells, and pH3^+^ cells in old midguts was significantly inhibited by eliminating ER stress in aged *Drosophila* (by overexpressing Ero1L or depleting of PEK; Ero1L and PEK are two key regulators of the ER stress in *Drosophila* (Koo et al., 2016)) (Figure 4h–p). Taurine supplementation could not further reduce the number of *esg*‐GFP^+^ cells, Dl^+^ cells, and pH3^+^ cells in *Drosophila* with a promoted UPR^ER^ activation induced by PEK depletion or Ero1L overexpression (Figure 4h–p). We also examined the effects of taurine and the UPR^ER^ on the gut digestive ability of aged flies. Consistent with previous results, eliminating ER stress in aged ISCs (achieved by either overexpressing Ero1L or depleting PEK) prevented the deterioration of gastrointestinal acid–base homeostasis, improved food intake and excretion of aged *Drosophila* (Figure 5a–c). Taurine supplementation was not able to further promote gut digestive function after eliminating ER stress (induced by Ero1L overexpression or PEK depletion) in aged files (Figure 5a–c).

Since the ER stress increases during aging (Salminen & Kaarniranta, 2010), we wanted to mimic this condition in young flies by depleting Xbp1 (a UPR^ER^ sensor (Wang, Zhu, et al., 2015; Wang et al., 2014)) or Ero1L (Wang, Zhu, et al., 2015) in ISCs to further explore the functional relationship between taurine and ER stress. As expected, the number of *esg*‐GFP^+^ cells, Dl^+^ cells, and pH3^+^ cells in young (14‐day‐old) midguts significantly increased and the lifespan dramatically decreased when Xbp1 (Figure 5d–i,m) or Ero1L (Figure 5j–l,m) were depleted. Taurine supplementation did not prevent this increase in these cell numbers (Figure 5d–l). Consistently, taurine did not rescue the phenotypes of decreased lifespan (Figure 5m), gastrointestinal acid–base homeostasis deterioration (Figure 5n), or declined digestive function in *Drosophila* with Xbp1‐ or Ero1L‐depleted ISCs (Figure 5o,p). These findings indicate that the ER stress functions downstream of taurine in preventing ISC hyperproliferation and the decline of digestive function in aged *Drosophila*.

### 2.7 Taurine prevents ISC hyperproliferation by counteracting ER stress‐associated JNK activity

**FIGURE 6:**
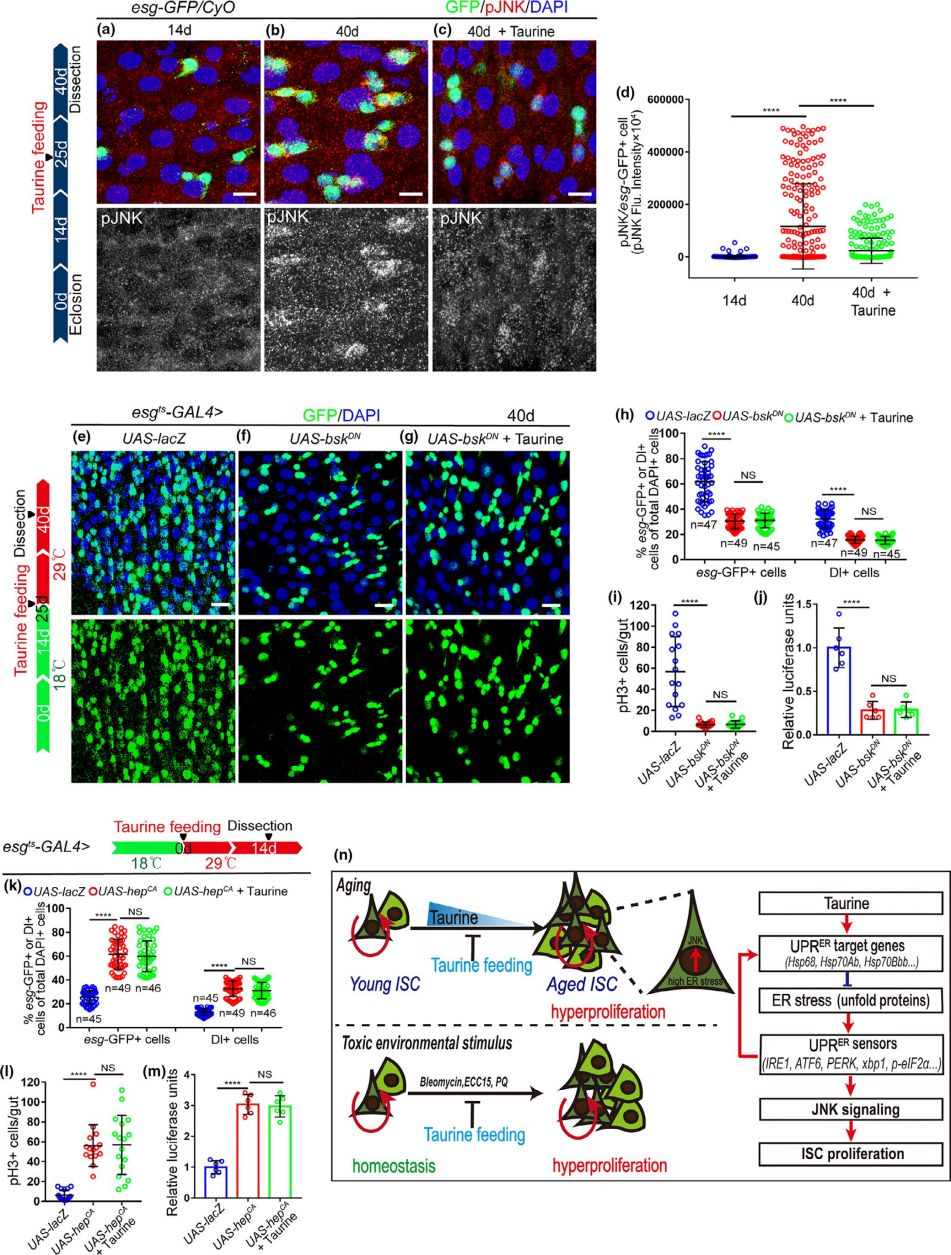
Taurine prevents ISC hyperproliferation by counteracting ER stress‐associated JNK activity. (a–c) Immunofluorescence imagines of pJNK (red) and *esg*‐GFP (green) staining of 14‐day flies, 40‐day flies, and 40‐day flies with taurine supplementation. Red arrows indicate pJNK‐positive cells. (d) Quantitation of pJNK intensity in esg‐positive cells from flies of 140‐day, 40‐day, and 40‐day with taurine supplementation. Each dot corresponds to one cell. (e–g) Immunofluorescence images of midguts of 40‐day flies with indicated genotypes and treatment, staining with *esg*‐GFP (green), Dl (red). (h, i) Quantification of the mean numbers of *esg*‐GFP^+^, Dl^+^, and pH3^+^ cells of flies with indicated genotypes and treatment. (j) Quantification of luciferase activity of midguts of flies with indicated genotypes and treatment. n indicates six independent experiments. (k, l) Quantification of the mean numbers of *esg*‐GFP^+^, Dl^+^, and pH3^+^ cells of flies with indicated genotypes and treatment. (m) Quantification of the luciferase activity in midguts with indicated genotypes and treatment. Error bars show the standard deviation (SD) of six independent experiments. (n) Schematic model of the mechanism. Taurine activates the UPR^ER^ target genes in ISCs, which in turn attenuates ER stress, prevents the over‐activation of JNK signaling, and inhibits the hyperproliferation of ISCs. Data information: Scale bars represent 10 µm. DAPI‐stained nuclei are shown in blue. Error bars represent SDs. Student's *t* tests, ****p < 0.0001. non‐significance(NS) represents *p* > 0.05

It has been reported that the UPR^ER^ target genes eliminate ER stress to suppresses ISC over‐proliferation by suppressing JNK signaling (Wang et al., 2014), so we investigated JNK activation in the ISCs of *Drosophila* in response to taurine administration by analyzing pJNK expression (an indicator of JNK signaling activation). Aged flies fed with taurine showed reduced activation of JNK signaling in ISCs (Figure 6a–d, and Figure S5a–c). Taurine administration did not further reduce the number of *esg*‐GFP^+^ cells, Dl^+^ cells, and pH3^+^ cells in *Drosophila* caused by the expression of a dominant‐negative version of *bsk* (*bsk^DN^*; *basket* (*bsk*) encodes a *Drosophila* c‐Jun N‐terminal kinase) (Figure 6e–j), which inhibits JNK signaling. As predicted, taurine supplementation did not rescue the increase in *esg*‐GFP^+^ cells, pH3^+^ cells, and Dl^+^ cells induced by *hep* ^CA^ (an active form of *hemipterous(hep)*; *hep encodes a Drosophila* JNK kinase) (Figure 6k–m). These results indicate that JNK functions downstream of taurine in regulating ISC homeostasis and suggests, therefore, that taurine prevents ISC aging by counteracting UPR^ER^‐associated JNK activity.

## 3 DISCUSSION

Although taurine, a conditionally essential amino acid, has been used to treat several aging‐associated diseases (Miyazaki & Matsuzaki, 2014), the underlying mechanism of taurine's role in anti‐aging remains largely unexplored. In this study, we showed that taurine abundance significantly decreases during aging and that in aged flies, taurine supplementation activates the UPR^ER^ target genes to eliminate ER stress in ISCs, retards midgut dysplasia, and promotes a healthy lifespan (Figure 6n).

Over the years, studies have shown taurine's various functions and mechanisms of action in organ development and it is essential for the development of the cardiovascular system, retina, and central nervous system (Wu, 2020). Other studies have shown that some nutrients for organ growth and development may also regulate organ aging, suggesting that there are common points between development and aging (Lee et al., 2015). Precise regulation of stem cell proliferation and differentiation is critical for organ development, and the dysregulation seen during aging is likely to result in malignant outcomes, including cancer. This study has shown that taurine, which is necessary for organ development, is also involved in the functional decline of stem cells in response to aging.

Bleomycin (BLM, causes DNA breaks and genomic instability) (Resende et al., 2017), *Erwinia carotovora* (*ECC15*, triggers a systemic immune response) (Ayyaz et al., 2015), and paraquat (PQ, induces oxidative stress) (Ayyaz et al., 2015) have been widely used to induce acute and chronic damage in *Drosophila* midguts, and simulate the environmental toxins that accelerate the aging process of *Drosophila*. In this study, we showed that taurine supplementation prevented the stimulus (BLM, *ECC15*, or PQ)‐induced midgut hyperplasia and increased the survival rate of flies under chronic damage (induced by continuously feeding with ECC15 and PQ). This suggests that taurine increases fly tolerance to noxious environmental stimuli. However, since oral administration of these toxins can cause systemic insults and taurine is also known to function beyond the intestine, we could not rule out the possibility that taurine supplementation might also benefit other tissues and cells increase the survival rate of flies under these chronic damages. Moreover, we should keep in mind that, although some of the basic pathways may be shared, the taurine supplementation may regulate different cellular responses to promote intestinal homeostasis and ISC health during the toxin (BLM, *ECC15*, or PQ) exposure and the natural aging. The exactly cellular responses to environmental toxin exposure and natural aging should be further studied.

The UPR^ER^ target genes attenuate endoplasmic reticulum (ER) stress caused by the accumulation of unfolded/misfolded proteins in the ER. Activation of the UPR^ER^ target genes is one of the strategies by which cells settle cellular metabolic error or environmental stimuli. The UPR^ER^ has been shown to regulate multiple cellular functions, including stem cell behavior (Guallar et al., 2020; Sigurdsson & Miharada, 2018; Tavasolian et al., 2020), and is emerging as a critical regulator of intestinal epithelium homeostasis (Wang, Zhu, et al., 2015). Recent studies in mice indicate that the UPR^ER^ may directly influence the gut regenerative process (Ma et al., 2017; Rosekrans et al., 2015) and in flies, it can modulate intestinal dysplasia in aged intestinal epithelium marked by excessive ISC proliferation (Wang, Zhu, et al., 2015). However, the underlying mechanisms of the UPR^ER^ regulation in ISC aging remain largely unexplored. This study demonstrates that taurine supplementation promotes the expression of UPR^ER^ target genes in ISCs which subsequently suppresses the JNK signaling pathway and has a protective role in ISC function and homeostasis. It is reasonable to presume that taurine may regulate other pathways that influence ISC aging and further studies are needed to uncover the details of how taurine modulates the expression of UPR^ER^ target genes during aging.

## 4 MATERIALS AND METHODS

### 4.1 Contact for reagent and resource sharing

Requests for further information, reagents, and resources should be directed to and will be fulfilled by the Lead Contact, Haiyang Chen (Chenhy82@scu.edu.cn).

### 4.2 *Drosophila* stocks and husbandry

*Drosophila* stocks were maintained at 25°C and fed with standard cornmeal fly food (for 1 L food: cornmeal 50 g, yeast 18.75 g, sucrose 80 g, glucose 20 g, agar 5 g, and propionic acid 30 mL). Adult UAS‐mediated gene knockdown or overexpression was accomplished through crossing with the *GAL4* lines in combination with the *GAL80^ts^* transgene at 18°C. The progeny were maintained at 29°C for indicated days to induce transgene expression. All flies used in this experiment were mated females. *Drosophila* lines used in this study are listed in Table S3.

### 4.3 Midgut dissection for RNA‐Seq

The midguts of adult flies were dissected in PBS by removing the foreguts, hindguts, Malpighian tubules, and trachea. Every sample contained at least 50 female midguts. Total RNA of midguts was prepared with TRIzol reagent (Invitrogen). Novaseq 6000 platform (Illumina, San Diego, US) and 150 bp paired ends were used to do the sequencing. 20 million reads per sample were obtained.

Low‐quality reads and adapter sequences were filtered out using Trimmomatic (v 0.39), and quality control were performed using FastQC (v0.11.8, http://www.bioinformatics.babraham.ac.uk/projects/fastqc/). Gene expression differences were analyzed using an R package (DESeq2), and KEGG pathway analysis was used to analyze the associated pathways.

### 4.4 FACS and qRT‐PCR

Each pooled sample contained midguts from a total of 100 mated females. These midguts were dissected and incubated with Elastase (Sigma, cat. no. E0258) for 1 h at 25°C with soft mixing every 15 min. The dissociated midguts were pelleted at 4°C at 400 g for 20 min and filtered through 70 µm filters. The *esg*‐GFP‐positive cells were sorted by flow cytometry (FACS Aria II sorter, BD Biosciences) with wild‐type flies (*w^1118^*) used as a negative control to set the background gate. For each biological replicate, about 40,000 *esg*‐GFP‐positive cells were collected and used for RNA‐seq. Total RNA was harvested using a commercial kit (Arcturus PicoPure RNA isolation kit, Applied Biosystems) and cDNA was synthesized using the PrimeScript RT reagent Kit (TaKaRa). The 2^−ΔΔCT^ method was used to calculate the expression of mRNA with Rp49 used as a housekeeping control. Primer sequences used for qPCR in this study are available upon reasonable request.

### 4.5 Immunofluorescence and microscopy

Female midguts were dissected and fixed with 4% EM‐grade paraformaldehyde fixation buffer (100 mM glutamic acid, 25 mM KCl, 20 mM MgSO4, 4 mM Na_2_HPO_4_, 1 mM MgCl_2_, pH 7.4) for 1 h at room temperature. The midguts were then washed with PBST (PBS plus 0.3% Triton X‐100) for 3 times, 10 min each, blocked with 5% BSA (in PBST buffer) for 30 min and incubated with primary antibody (dissolve in PBST buffer) at 4°C overnight. Then, they were washed and incubated with corresponding secondary antibodies and DAPI for 2 h at room temperature. The primary antibodies used are listed in the Reagent Table (Table S4). All images were obtained using a Leica TCS‐SP8 confocal microscope. Adobe Photoshop and Adobe Illustrator were used to assemble the images.

### 4.6 Luciferase assays

The Firefly Luciferase Reporter Gene Assay Kit (Beyotime Biotechnology, Jiangsu, China, RG051S) was used to measure luciferase activity. Each sample contained about 15 female midguts and was immediately frozen with liquid nitrogen. To each sample, 50 µl of the Luciferase Reporter Gene Assay Lysis Buffer was added and the sample was homogenized. Extracts were obtained by centrifugation at 13,000 *g* for 10 min at 4°C. Luciferase activity was measured according to the manufacturer's instructions.

### 4.7 Bromophenol blue assay

The bromophenol blue assay was used to detect the pH change in the midgut as previously described (Li et al., 2016). Briefly, 100 µl of 2% Bromophenol blue sodium (Sigma, B5525) was added to the food surface and several holes were poked to allow the Bromophenol blue solution completely absorbed by the food. Images were taken immediately after 12 h.

### 4.8 Cafe assay and fly excretion measurements

The Café and excretion measurements were performed as previously described (Deshpande et al., 2014). Briefly, two capillaries (53432‐706, VWR) containing 5 μl of liquid food were used and food consumption‐ability was calculated from volume reduction.

To rule out the possibility that taurine affects the food preference or food intake behavior of *Drosophila*, we designed one kind of modified *Drosophila* vials (each of these vials contains 6 liquid food‐contained capillaries, 3 with taurine, and 3 without taurine, see the cartoon in Figure S3b) where flies can freely choose taking or not taking taurine‐contained liquid food.

### 4.9 Solid food intake measurements

The solid food intake measurements were performed as previously described (Shell et al., 2018). Briefly, an agar‐based food medium containing Blue 1 (FD and C Blue No. 1, Spectrum Chemical Manufacturing Corp, FD110) was prepared at a concentration of 1% (wt/vol) by using plastic feeder caps. Adult flies (15/vial) in the vials consumed medium from the feeder caps and then excreted waste over time. The dye excreted by flies on the walls of the vials (excreted vial dye, ExVial) and the dye inside the flies (internal dye, INT) were collected. The spectrophotometer was used to determine the absorbance (Wavelengths, 630 nm) of the INT and ExVial dye in water extracts.

### 4.10 “Smurf” assay

The “Smurf” assays were performed as previously described (Rera et al., 2011, 2012). Briefly, the medium of Blue dye no. 1 (FD and C Blue No. 1, Spectrum Chemical Manufacturing Corp, FD110) was prepared using the standard medium with dyes added at a concentration of 2.5% (wt/vol). Flies (20/vail) were maintained on Blue dye no. 1 medium. After 9 h, the fly was counted as a Smurf when the coloration of Blue dye no. 1 could be observed outside of the digestive tract.

### 4.11 Taurine, Paraquat, Bleomycin, and *ECC15* treatment

Taurine was dissolved in DMSO and mixed with regular food medium. Female flies were collected no more than 3 days after eclosion and distributed equally into vials containing taurine mixed food. The control food was mixed with an equal volume of DMSO. Chromatography paper was cut into 3.7 × 5.8 cm strips and saturated with 10 mM paraquat (Aladdin, M106760) or 25 µg/ml bleomycin (Aladdin, B107423). For *ECC15* treatment, a concentrated bacterial pellet was centrifuged from overnight culture media and was dissolved in 1 ml 5% sucrose (to make the final OD_600_ = 200). Before treatment, flies were starved in empty vials for 1 h and transferred into vials with paraquat, bleomycin, or *ECC15* saturated paper. After 24 h of treatment, flies were transferred daily to new vials with paraquat, bleomycin, or *ECC15*. And 5% (wt/vol) sucrose without those drugs was used as controls.

### 4.12 Liquid chromatography–electrospray ionization–mass spectrometry (LC‐MS) analysis

Chemicals and reagents, taurine (T103829), 2‐Ethylbutyric acid (an internal standard for taurine, E105668), ammonium formate (A100185), and formate (F112034), were obtained from Aladdin (Shanghai, China). The acetonitrile (ACN) and methanol of chromatography grade were obtained from Sigma‐Aldrich. For taurine extractions, female midguts were dissected by removing Malpighian, trachea, foreguts, and hindguts. For each condition, three independent samples were collected from mated females. The detailed process of the LC‐MS analysis was as described previously (Du et al., 2020). Briefly, 500 µl methanol was used to extract taurine. All LC‐MS analysis was performed with a TripleTOFTM 5600+ (AB, USA). HPLC (SHIMADZU, LC20A) with Poroshell 120 LC was used to perform liquid chromatography. Mobile phase A: 10 mmol/L ammonium formate (0.05% formate). Mobile phase B: ACN. The ratio of A: B is 1: 9. The flow rate is 0.1 ml/min. The concentration of taurine was calculated by normalizing the peak area of taurine concerning the internal standard. The parameter of taurine is m/z 124.0‐> 80.

### 4.13 Lifespan assays under normal and stressful conditions

For survival tests under normal conditions, 100 female or male unmated flies (1‐ to 2‐day‐old) of the same genetic background were collected and distributed equally into four vials with regular food medium with or without taurine medium supplementation. Flies that were still alive were counted every 2 days. The viability tests were repeated as three independent experiments.

For survival tests under stressful conditions, flies were divided into four groups which are paraquat (20 mM) group, paraquat (20 mM) added taurine group, *ECC15* (OD_600_ = 200) group, and *ECC15* (OD_600_ = 200) added taurine group. For each group, 100 female flies (1‐ to 2‐day‐old) of the same genetic background were collected and distributed equally into four vials. At the same time, 10 male flies (1‐ to 2‐day‐old) were added to each vial to ensure that the females were mated. Female flies that were still alive were counted every day.

### 4.14 Quantification and statistical analysis

GraphPad Prism v6.0 was used to evaluate statistical significance after verifying normality and equivalence of variances. For stem cell counts, means ± SD was displayed. Student's two‐tailed t tests and log‐rank test were performed. *p* < 0.05 was considered to be statistically significant. All experiments were repeated at least three times.

## CONFLICT OF INTEREST

The authors declare no competing interests.

## AUTHOR CONTRIBUTIONS

Conceptualization: H.C.; Methodology: G.D., Z.L., Z.Z., and H.C.; Investigation: G.D., Y.L., and Z.L; Writing–Original Draft: Z.Y., G.D., Z.L., and H.C.; Writing—Review and Editing: G.D., Z.Y., Z.L., Y.Z., J.Z., and H.C.; Funding Acquisition: H.C.; Resources: H.C.; Supervision: H.C.

### OPEN RESEARCH BADGES

The RNA‐seq data that support the findings of this study have been deposited in the Sequence Read Archive (SRA) under BioProject ID PRJNA644243 (https://www.ncbi.nlm.nih.gov/sra/?term=PRJNA644243). Source data for Figure 4 have been provided as Supplementary Table S1 and Supplementary Table S2. RNA‐seq data were analyzed using R software (version 3.5.3) obtained from https://www.r-project.org/.

## Supporting information

Fig S1

Fig S2

Fig S3

Fig S4

Fig S5

Table S1

Table S2

Table S3

Table S4

## Data Availability

The authors declare that all data generated or analyzed during the current study are available from the corresponding author upon reasonable request.
