# Supplementary figures and images for: Taurine represses age‐associated gut hyperplasia in Drosophila via counteracting endoplasmic reticulum stress
Source: Aging Cell. 2021 Feb 9;20(3):e13319. doi: 10.1111/acel.13319 (PMC7963329; doi:10.1111/acel.13319)

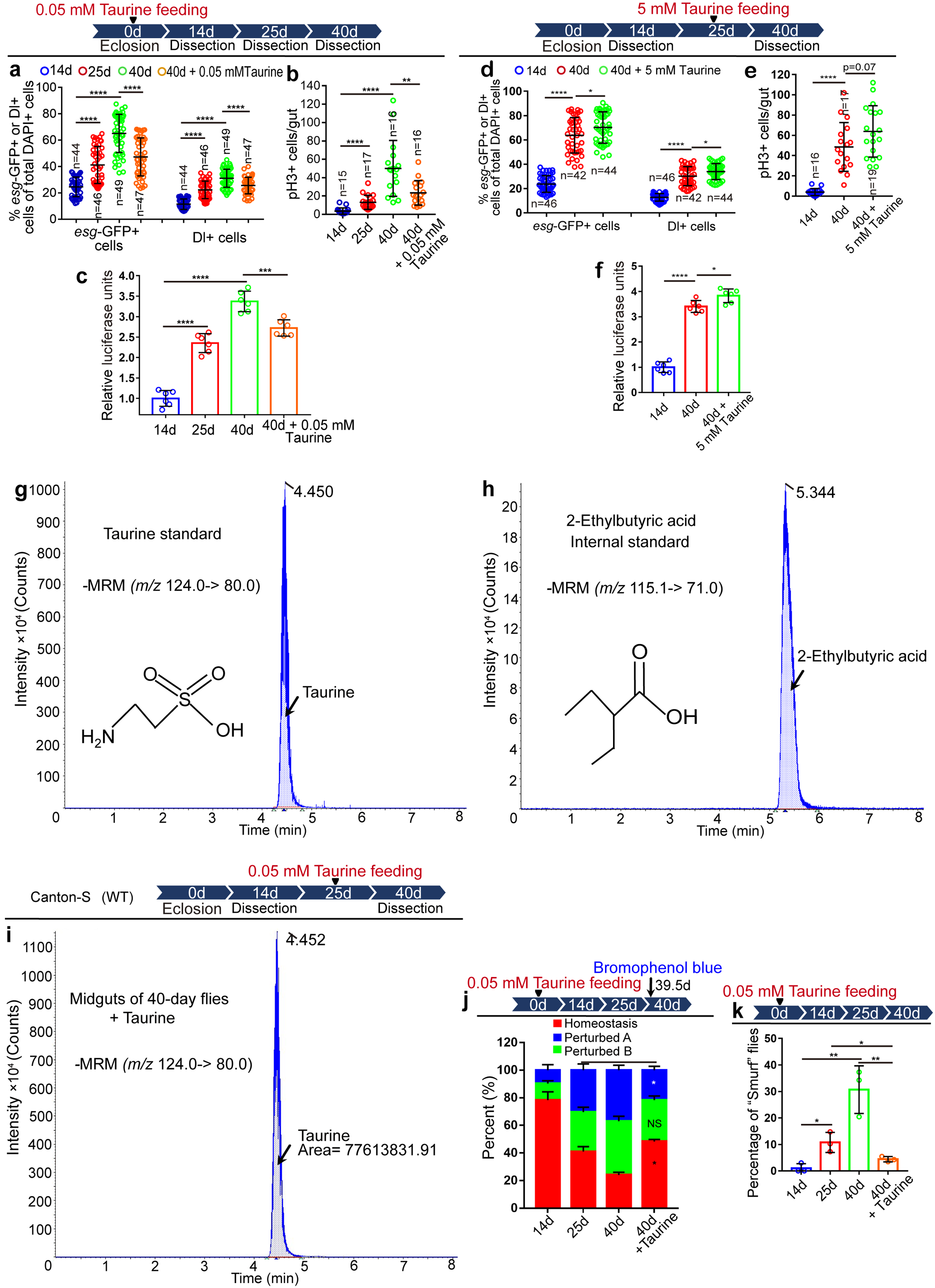

Supplement: Supplementary file 1 — Fig S1 [file ACEL-20-e13319-s002.tif]

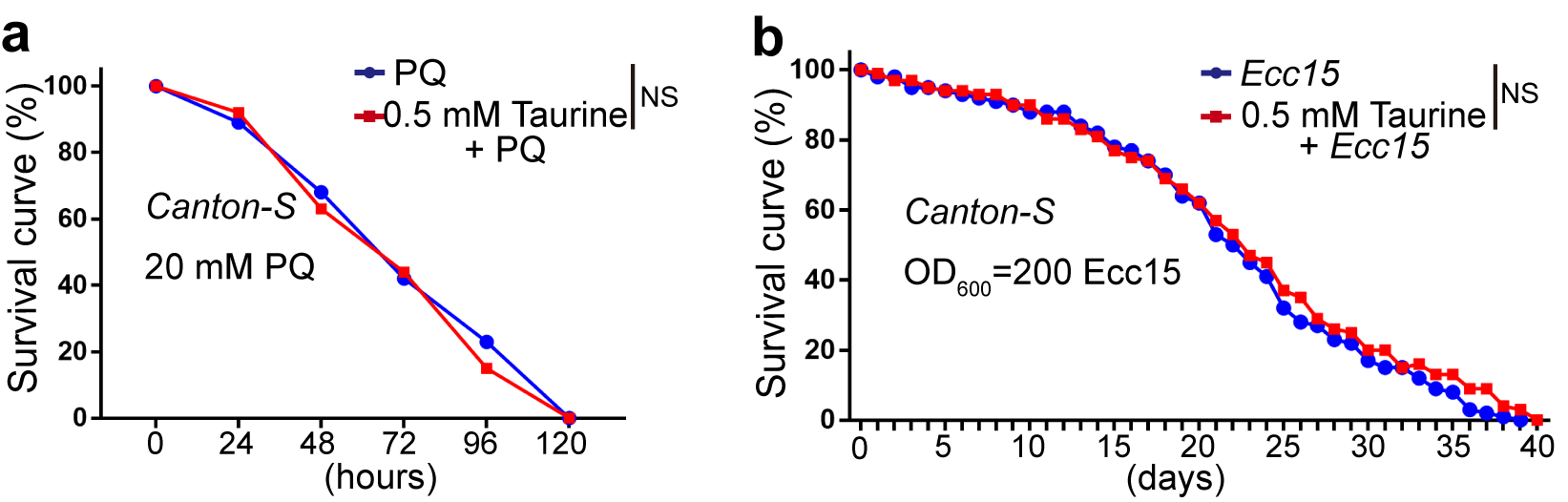

Supplement: Supplementary file 2 — Fig S2 [file ACEL-20-e13319-s007.tif]

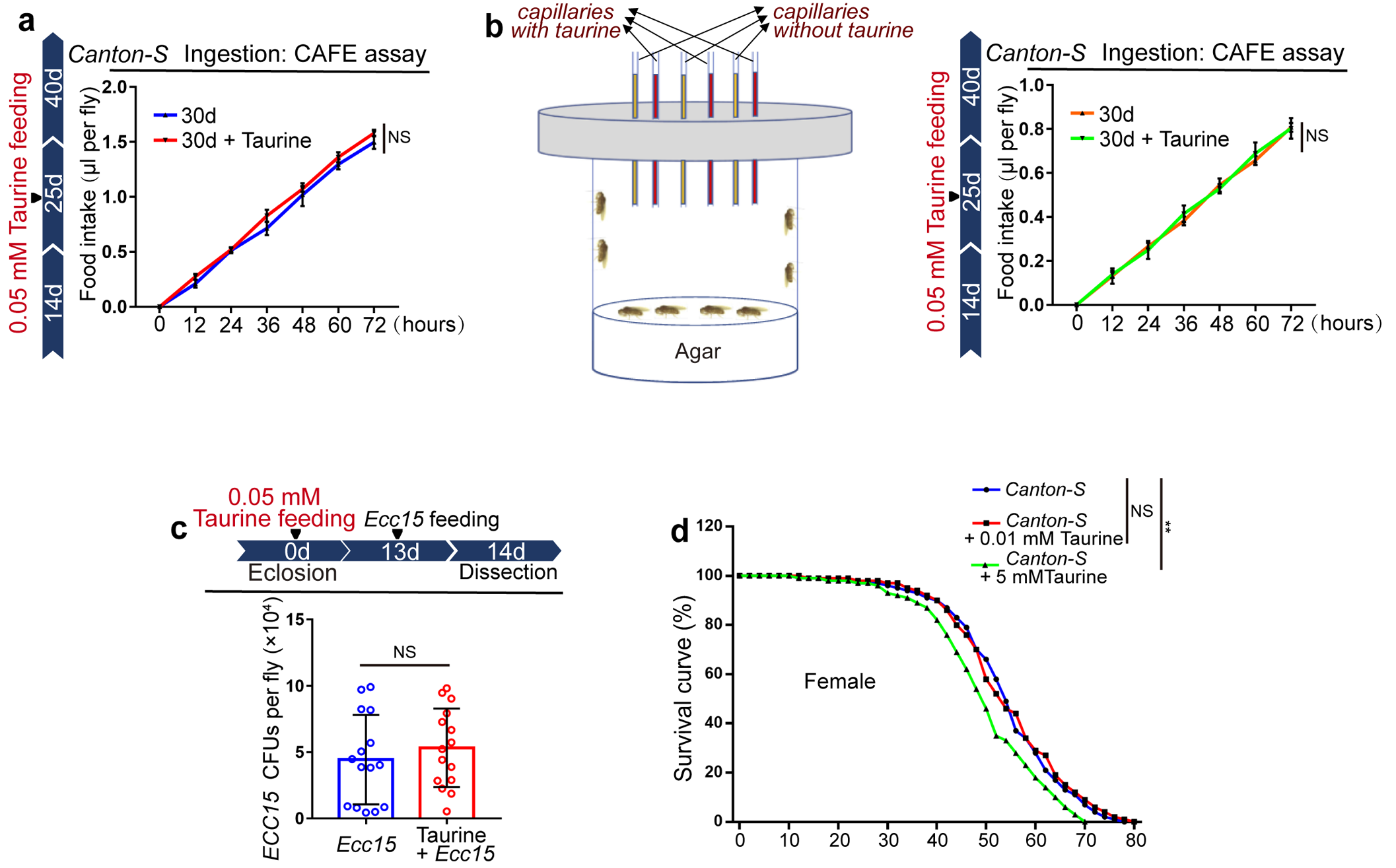

Supplement: Supplementary file 3 — Fig S3 [file ACEL-20-e13319-s004.tif]

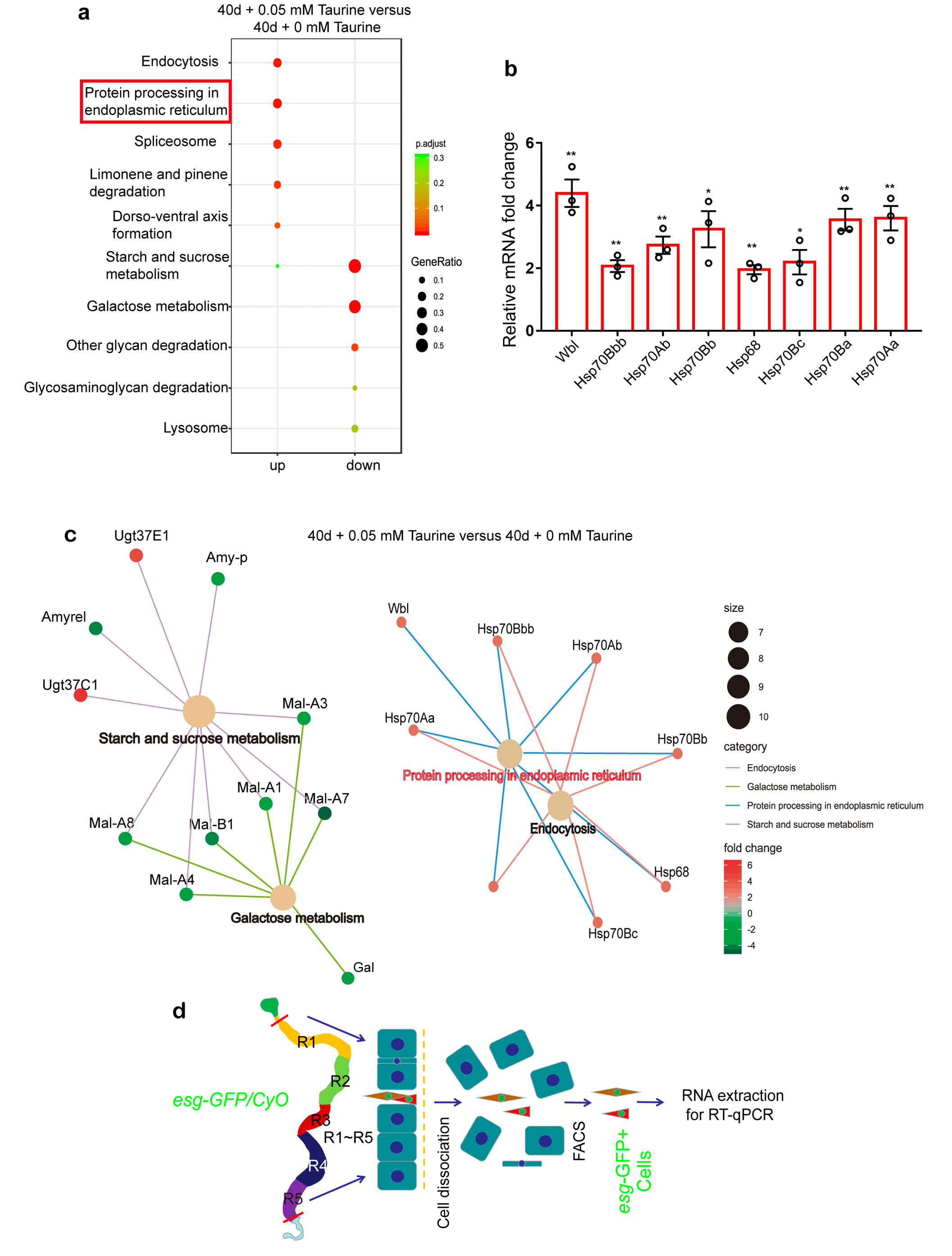

Supplement: Supplementary file 4 — Fig S4 [file ACEL-20-e13319-s008.tif]

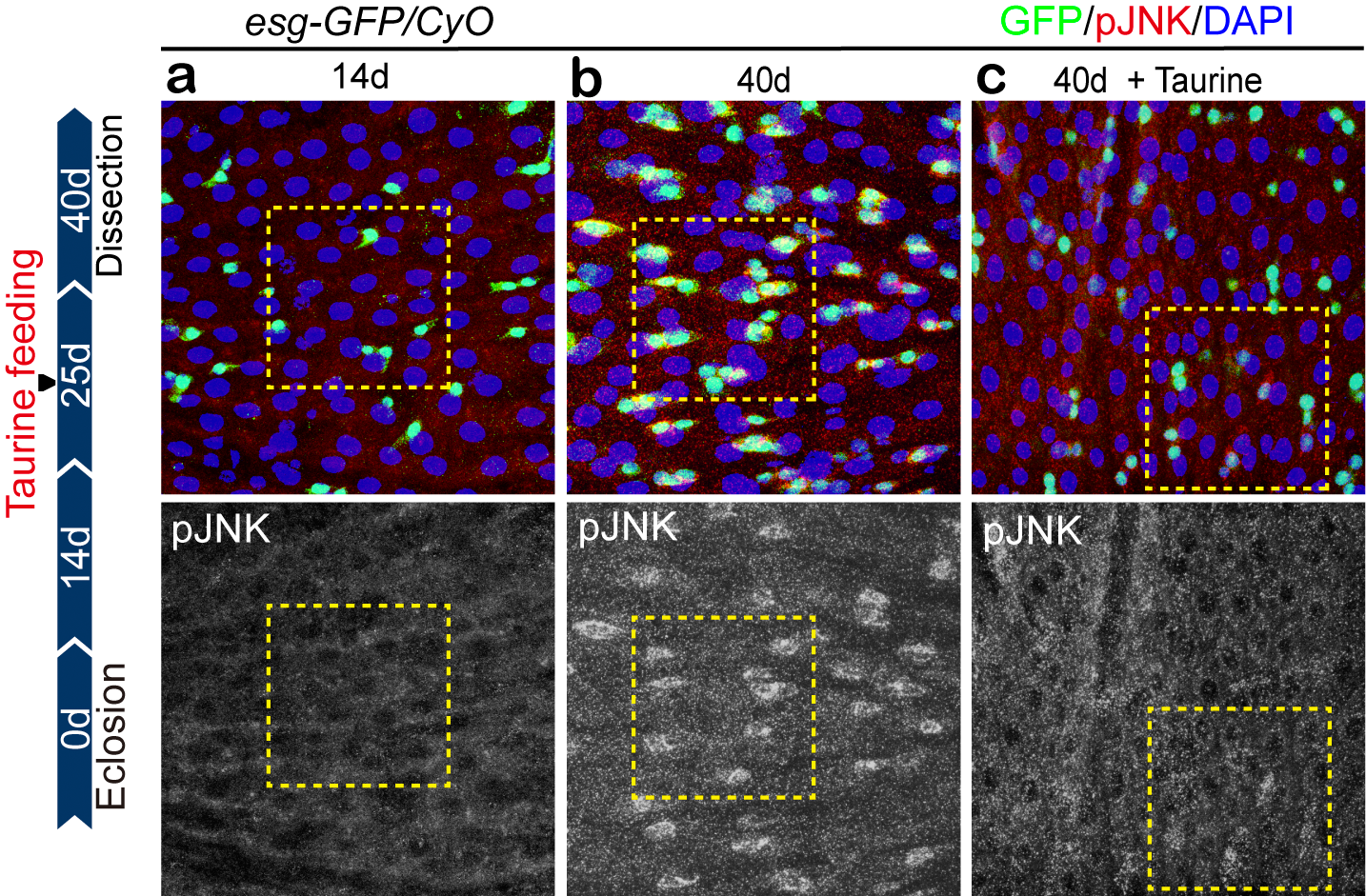

Supplement: Supplementary file 5 — Fig S5 [file ACEL-20-e13319-s001.tif]
